# Membrane Activity of LL-37 Derived Antimicrobial Peptides against *Enterococcus hirae*: Superiority of SAAP-148 over OP-145

**DOI:** 10.3390/biom12040523

**Published:** 2022-03-30

**Authors:** Paulina Piller, Heimo Wolinski, Robert A. Cordfunke, Jan Wouter Drijfhout, Sandro Keller, Karl Lohner, Nermina Malanovic

**Affiliations:** 1 Biophysics, Institute of Molecular Biosciences (IMB), University of Graz, Humboldstr. 50/III, 8010 Graz, Austria; paulina.piller@uni-graz.at (P.P.); sandro.keller@uni-graz.at (S.K.); karl.lohner@uni-graz.at (K.L.); 2 Field of Excellence BioHealth, University of Graz, 8010 Graz, Austria; heimo.wolinski@uni-graz.at; 3 Biochemistry, Institute of Molecular Biosciences (IMB), University of Graz, Humboldstr. 50/II, 8010 Graz, Austria; 4 Department of Immunology, Leiden University Medical Center, 2300 RC Leiden, The Netherlands; r.a.cordfunke@lumc.nl (R.A.C.); j.w.drijfhout@lumc.nl (J.W.D.); 5 BioTechMed-Graz, 8010 Graz, Austria

**Keywords:** antimicrobial peptides (AMPs), membrane-peptide interaction, antimicrobial activity, phospholipids, membrane permeability, membrane depolarization, electrostatic interaction, phosphatidylglycerol (PG) and cardiolipin (CL)

## Abstract

The development of antimicrobial agents against multidrug-resistant bacteria is an important medical challenge. Antimicrobial peptides (AMPs), human cathelicidin LL-37 and its derivative OP-145, possess a potent antimicrobial activity and were under consideration for clinical trials. In order to overcome some of the challenges to their therapeutic potential, a very promising AMP, SAAP-148 was designed. Here, we studied the mode of action of highly cationic SAAP-148 in comparison with OP-145 on membranes of *Enterococcus hirae* at both cellular and molecular levels using model membranes composed of major constituents of enterococcal membranes, that is, anionic phosphatidylglycerol (PG) and cardiolipin (CL). In all assays used, SAAP-148 was consistently more efficient than OP-145, but both peptides displayed pronounced time and concentration dependences in killing bacteria and performing at the membrane. At cellular level, Nile Red-staining of enterococcal membranes showed abnormalities and cell shrinkage, which is also reflected in depolarization and permeabilization of *E. hirae* membranes. At the molecular level, both peptides abolished the thermotropic phase transition and induced disruption of PG/CL. Interestingly, the membrane was disrupted before the peptides neutralized the negative surface charge of PG/CL. Our results demonstrate that SAAP-148, which kills bacteria at a significantly lower concentration than OP-145, shows stronger effects on membranes at the cellular and molecular levels.

## 1. Introduction

Membrane disruption mediated by antimicrobial peptides (AMPs) represents an alternative strategy to eradicate bacteria more efficiently than conventional antibiotics [1]. Such a non-specific mode of action caused by a disruption of fundamental barrier function [2,3,4,5] leads to cell death within minutes and a killing rate faster than the bacterial growth rate, which makes resistance less likely to occur [6]. As effector molecules of the innate immunity [5] AMPs are widely conserved through humans, animals, plants, bacteria, fungi, protists, and archaea [7,8]. Their research in the antimicrobial field markedly expanded with more than 3000 peptides of natural or synthetic origin reported to date by the AMP Database [7]. However, only a few peptides thus far have been used in clinical trials.

**Figure 1:**
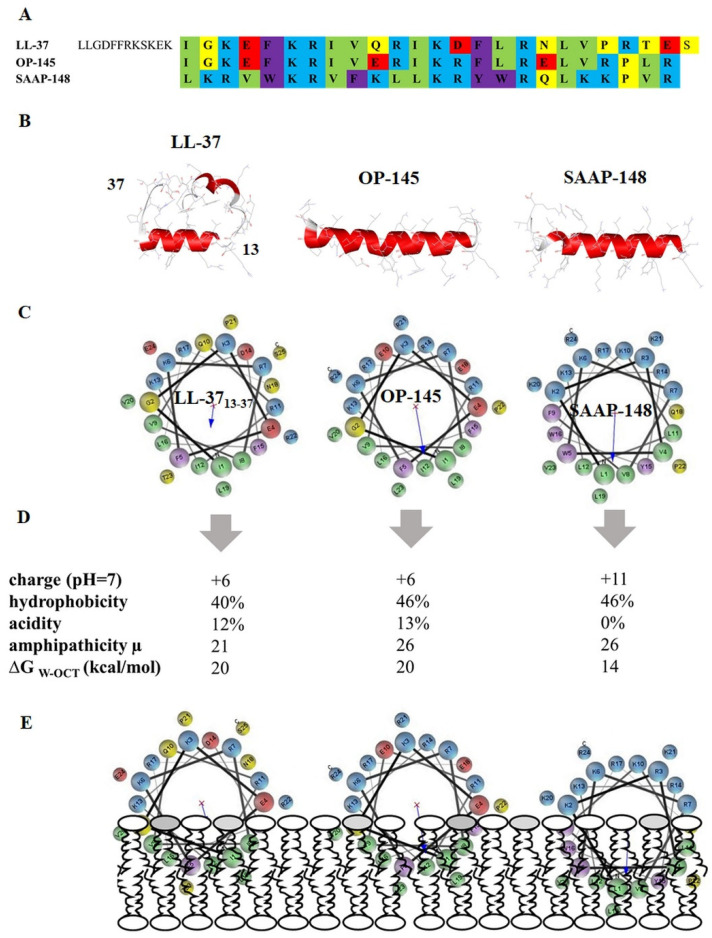
Structural properties of AMPs. (**A**) amino acid sequence. Cationic residues are shown in blue, anionic in red, aromatic in purple, aliphatic in green, and polar in yellow; (**B**) PEP-FOLD alpha-helix prediction obtained from mobile server (https://mobyle.rpbs.univ-paris-diderot.fr/cgi-bin/portal.py#forms::PEP-FOLD); (**C**) helical-wheel projection provided by Membrane Protein Explorer MPEx (https://blanco.biomol.uci.edu/mpex/); (**D**) charge, hydrophobic and acidic residues, Wimley–White partitioning parameters (amphipathicity = hydrophobic moment, µ; ∆G_W-OCT_, free energy transfer from water to bilayer); and (**E**) schematic illustration of the proposed partitioning of the peptides into the membrane.

Several reports summarize limitations of AMPs as therapeutic agents mainly focusing on their bio-incompatibility, safety, high production costs, or inefficient activity in respective environment and lack of superiority over conventional treatment [9,10]. Usually, further evaluation of the peptide sequence led to design of novel peptides with improved activities. The same was applied to human cathelicidin LL-37 [3,11,12,13], one of the most studied AMPs which, amongst others, due to its proteolytic instability was not suitable for further clinical studies. Indeed, development of OP-145 by removing the proteolytically instable N-terminal part of the first 12 residues (Figure 1) led to improved activity compared with LL-37 [13]. Although OP-145 exhibited valuable antimicrobial activity against pathogenic bacteria such as methicillin-resistant *Staphylococcus areus* (MRSA) [11,13,14,15] and has proven to efficiently treat chronic otitis in patients [16], clinical phase II trials were not continued. Follow-up modifications resulted in a peptide, termed SAAP-148 [3] (Figure 1). Interestingly, SAAP-148 possesses an activity profile better than that of any other AMP, which is currently under preclinical investigation [3,17]. Until now, some peptides, such as nisin, daptomycin, and polymyxin, have been approved for clinical use, but they all have specific membrane targets, such as lipopolysaccharides or phosphatidylglycerol and, therefore, are active against only one of the bacterial classes. However, compared with classical AMPs, these peptides have different structural properties, being circular or negatively charged in the case of daptomycin. LL-37 derived peptides are linear alpha-helical peptides and exhibit a wide spectrum of bactericidal activities against Gram-positive as well as Gram-negative bacteria including biofilms [3,11,15]. This points to a more general mechanism, which might cover a broad range of bacterial envelopes.

Different kinds of modes of action have been proposed for LL-37, ranging from classical membrane disruption into small vesicles to pore-forming mechanisms [18,19]. These conclusions were drawn mainly from experiments on simple model systems—that is, lipid-only systems resembling the bacterial cytoplasmic membrane—which do not account for initial interactions with a bacterial cell such as its surface or cell-wall components, e.g., peptidoglycan (PGN), LPS, or (lipo)-teichoic acid (LTA/TA). Basically, explanations included the following arguments: depending on the lipids’ and peptide’s nature (for review see [20]), the association of the peptides with lipids destabilizes the membrane and leads to a loss of membrane integrity and final collapse of the membrane. Major characteristics of the peptides and bacterial components, which strongly affect the antimicrobial activity [8,15,20], especially in selecting between different bacteria through binding to different targets [2,5,21] involves their capability for electrostatic and hydrophobic interaction. Besides the overall amphipathicity, other physicochemical properties, such as H-bonding capacity and structural flexibility are important.

Earlier studies from our group showed that the parent peptide LL-37 [22,23], as well as OP-145 [15], interact differently with the phospholipid (PL) matrix. Interactions of LL-37, OP-145, as well as SAAP-148, with one of the major bacterial PLs, phosphatidylglycerol (PG), result in a thinning of the bilayer, which is further associated with permeabilization of such membranes [3,15,23]. At the cellular level, it has been shown that SAAP-148 permeabilizes the cytoplasmic membrane of Gram-positive bacterium *S. aureus* as well as of the Gram-negative bacterium *A. baumannii* [3]. Further, cryo-electron micrographs of *S. aureus* exposed to SAAP-148 clearly showed ruptured membrane zones. In some areas, invaginations of cytoplasmic membranes were observed, whereby the cell wall remained always intact. Electron micrographs of LL-37 treated bacteria [24,25,26] showed damaged membranes, but, primarily, effects on the cell wall of *E. coli* [25] as well as *S. aureus* [26]. Recently, LL-37 has been crystalized in the presence of detergents, and a structure of a narrow tetrameric channel with a strongly charged core was observed pointing to pathways for the passage of water molecules [24]. However, it is unclear how formation of such a channel in bacteria might induce marked cell wall lysis and formation of extracellular vesicles observed for bacteria exposed to LL-37 [24,25].

In this study, we aimed to assess differences in the mode of action of OP-145 and SAAP-148 on the basis of their physicochemical properties (Figure 1). Both peptides have the same length of 24 amino acid residues, whereby at physiological pH SAAP-148 has a higher net charge (+11) as compared with OP-145 (+6). In addition, SAAP-148 does not contain any acidic residues. Although the total hydrophobicity and amphiphathicity did not differ between the peptides, helical wheel projections indicate a larger hydrophobic region in the SAAP-148 sequence (Figure 1C). In silico studies of membrane partitioning revealed that SAAP-148 needs less free energy to transfer from water to the hydrophobic area, the bilayer. In this context, higher membrane destabilization could be expected as SAAP-148 can penetrate more easily and deeper into the membrane (Figure 1E). Thus, we tested the potential of these two peptides for membrane activity on Gram-positive *E. hirae* and model membranes composed of the major PLs of enterococcal membranes, PG and cardiolipin [27].

## 2. Experimental Procedures

### 2.1. In Silico Evaluation of the Peptide’s Structural Properties

Primary single amino acid sequences of the peptides were uploaded on an online tool available at PEP-FOLD server @ the RPBS Mobyle Portal (https://bioserv.rpbs.univ-paris-diderot.fr/services/PEP-FOLD/), whereby all atoms were generated by applying the coarse-grained force field approach [28,29]. Generated models for prediction of 3D secondary structure were clustered using Apollo [30]. The basic structural parameters, hydrophobicity, acidity, and charge of the peptides were calculated by the peptide hydrophobicity/hydrophilicity analysis tool from Peptide 2.0 (https://www.peptide2.com/N_peptide_hydrophobicity_hydrophilicity.php). Helical wheel projections, hydrophobic moment (µ), and bilayer partitioning free energy (ΔG) were obtained from Totalizer, a tool provided by Membrane Protein Explorer, mPEX ([31], https://blanco.biomol.uci.edu/mpex/). Totalizer calculates the hydropathy plot of the peptides based on whole-residues hydrophobicity Wimley–White scales [32]. This allows evaluation of the exact costs of partitioning the H-bonded peptide bonds of alpha-helices into the membrane. Bilayer partitioning free energy was measured using the octanol scale, describing residue’s free energy of transfer from water to bilayer hydrophobic core/to octanol.

### 2.2. Materials

Unless otherwise indicated, all solutions were prepared in Napi buffer pH 7.4, containing 20 mM NaPi and 130 mM NaCl. NaCl and disodium phosphate (water-free) were obtained from Carl Roth and disodium phosphate monohydrate from Merck (Vienna, Austria). For leakage assays, HEPES buffer pH 7.4 was used; composed of 10 mM HEPES and 140 mM NaCl. The 4-(2-Hydroxyethyl) piperazine-1-ethanesulfonic acid (HEPES, ≥99.5%) was acquired from Sigma-Aldrich (Vienna, Austria). PLs (>99% purity) 1-palmitoyl-2-oleoyl-*sn*-glycero-3-phospho--1’-*rac*-glycerol) (POPG; MW = 771 g/mol), 1,2-dimyristoyl-*sn*-glycero-3-phospho-(1’-*rac*-glycerol) (DMPG; MW = 689 g/mol), 1’,3’-bis[1,2-dioleoyl-*sn*-glycero-3-phospho]-glycerol (TOCL; MW = 1808 g/mol) and 1’,3’-bis[1,2-dimyristoyl-*sn*-glycero-3-phospho]-glycerol (TMCL; MW = 1275 g/mol) were purchased from Avanti Polar Lipids (Alabaster, AL, USA). The peptides OP-145 and SAAP-148 were synthesized as described previously [3,15]. Peptide purity was >95%. *E. hirae* ATCC10541 was obtained from LGC Standards (Wesel, Germany).

### 2.3. Methods

#### 2.3.1. Growth Conditions of *Enterococcus hirae*

For overnight cultures, Brain-Heart-Infusion Broth (BHIB) (Carl Roth, Graz, Austria; Beef heart 5 g/L, calf brains 12.5 g/L, disodium hydrogen phosphate 2.5 g/L, D (+)-glucose 2g/L, peptone 10 g/L, NaCl 5 g/L, final pH 7.4 ± 0.2 (25 °C)) was inoculated with *E. hirae* cells of a single colony placed at 37 °C under shaking conditions overnight. After that, the main culture was inoculated starting with OD = 0.01 and incubated in fresh BHIB medium to mid-logarithmic phase for 3 h 30 at 37 °C by shaking (OD is approximately 1).

#### 2.3.2. Preparation of Model Membranes

Lipid films were prepared by using an appropriate amount of PL dissolved in chloroform/methanol (9:1 *v*/*v*) (Carl Roth, Graz, Austria), followed by evaporation under a stream of nitrogen for 30 min at 35 °C and storage in vacuum overnight [15]. After the addition of NaPi buffer or fluorophore buffer for leakage assays, lipid vesicles were formed by intermittent vigorous vortexing at 65 °C. For ζ-potential measurements or leakage assays, large unilamellar vesicles (LUVs) were obtained by extrusion of multilamellar vesicles through a polycarbonate filter (Millipore-Isopore, Sigma Aldrich, Vienna, Austria) with a pore size of 0.1 µm. Vesicle size was measured using a Zetasizer Nano (ZSP, Malvern Panalytical, Prager Electronics, Wolkersdorf, Austria)).

#### 2.3.3. Antimicrobial Activity of OP-145 and SAAP-148

After culturing the cells in BHIB, they were washed once with NaPi buffer. 1 × 10^6^–1 × 10^9^ CFU/mL *E. hirae* in NaPi buffer were incubated for 15 min with the defined peptide concentration. Antimicrobial activity can be determined by plating a 100-µL sample on diagnostic-sensitivity Brain-Heart-Infusion agar (BHIB + 2 w% agar (Carl Roth, Graz, Austria)) and subsequent establishing of the number of viable bacteria after incubation at 37 °C overnight. The 99.9% lethal concentration (LC_99.9%_) is defined as the lowest peptide concentration that killed ≥99.9% of bacteria.

#### 2.3.4. Depolarization of *E. hirae* Membrane and Fluorescence Microscopy

Membrane depolarization, similar to [33], was performed by using *E. hirae* cells in mid-logarithmic phase, which were collected by centrifugation, washed twice, and then resuspended in NaPi buffer to an OD of 0.0125 (1 × 10^7^ CFU/mL). To investigate the cytoplasmic membrane depolarization by OP-145 and SAAP-148 1 µM cyanine dye DiSC_3_(5) were applied. To allow dye uptake, cells were incubated for 30 min in the dark. Before the measurement, 100 mM KCl was added. The desired AMP was added gradually to yield a final concentration of 51.2 µM peptide. As positive controls, 3.5 µM Melittin (Sigma-Aldrich, Vienna, Austria) and 1% Triton 100 (Carl Roth, Graz, Austria) were used. The fluorescence intensity was recorded on a VARIAN Cary Eclipse Fluorescence Spectrophotometer at an excitation wavelength of 622 nm and an emission wavelength of 685 nm with a bandwidth of 5 nm. High-precision QS cuvettes (10 mm × 10 mm) with a volume of 2 mL (Helma Analytics, Sigma Aldrich, Vienna, Austria) were used for all measurements.

#### 2.3.5. Membrane Permeabilization of *E. hirae* and Flow Cytometry

Cells were washed with NaPi buffer twice (5 min, 5100× *g*), diluted to 1 × 10^6^ CFU/mL with NaPi and stored on ice. In total, 600–1200 µL of the cell suspension were incubated with propidium iodide (PI, 1 µg/mL final concentration) followed by the fluorescence measurement in polystyrene tubes with the flow cytometer BD LSR Fortessa using the BD FACSDiva Software (Version 8.0.1, Beckton Dickinson, San Hose, CA, USA; excitation: 488 nm, emission: 695/40 nm, PerCP-Cy5.5 channel). To distinguish between PI-positive and PI-negative cell populations, selective gating was used. The scan rate was at 300–400 events per second. After 30 s, the appropriate peptide amount has been added to the labeled bacterial cells, followed by the fluorescence measurement for 10 min for OP-145 and SAAP-148 treated cells. For the analysis of PI-positive cells, the fcs-files from the BD FACSDiva Software had to be extracted into csv-files by using open-source FCSExtract Utility (version 1.02). The percentage of PI-positive cells was calculated at different time intervals in Microsoft Excel.

#### 2.3.6. Staining of *E. hirae* Membranes and Fluorescence Microscopy

*E. hirae* cells were washed with NaPi buffer twice (5 min, 5100× *g*) and diluted to 2.5 × 10^8^ CFU/mL. The incubation of the cells with the appropriate peptide concentration was accomplished for 10 min at RT. Afterwards, the cells were treated with Nile Red (Sigma Aldrich, Vienna, Austria.) for 1 min at RT (final concentration: 10 µg/mL). Subsequently, the cells were centrifuged and 2 µL of the cell pellet was mounted on an agar-coated microscope slide for cell immobilization [34].

Microscopy was performed using a Leica SP5 confocal microscope (Leica Microsystems, Vienna, Austria) with spectral detection and a Leica HCX PL APO CS 63x NA 1.4 oil immersion objective at 47 × 47 nm sampling (x/y). Nile Red was excited at 561 nm, fluorescence emission detected between 570 to 750 nm. Fluorescence and transmission images were recorded simultaneously.

#### 2.3.7. Permeability of Model Membrane and Fluorescence Spectroscopy

As previously described [15,35], leakage of the aqueous content from LUVs composed of POPG/TOCL (80:20 mol/mol) upon incubation with the peptide was determined by using 20 mg/mL lipid vesicles of defined size loaded with 8-aminonaphthalene-1,3,6,-trisulfonic acid/*p*-xylene-bis-pyridinium bromide (ANTS/DPX). Separation of LUVs from free fluorescent dye was performed by size exclusion chromatography using a column filled with Sephadex G-75 (Amersham Biosciences, Vienna, Austria) fine gel swollen in an iso-osmotic buffer (20 mM HEPES, 140 mM NaCl, pH 7.4). The PL concentration was determined as described below. Fluorescence emission spectra were obtained using an excitation wavelength of 360 nm, an emission wavelength of 530 nm, and a bandwidth of 10 nm. The fluorescence measurements were performed in quartz cuvettes (10 mm × 10 mm, high-precision cells, Helma Analytics, Sigma Aldrich, Vienna, Austria) with an initial lipid concentration of 50 µM in 2 mL HEPES buffer. Fluorescence emission was recorded as a function of time before and after the addition of gradual amounts of the peptide. Because of leakage and subsequent dilution of dye, the observed fluorescence increase was measured after peptide addition ranging from 0.25 µM up to 8 µM, which corresponds to 0.5 to 16 mol% peptide. The measurements were carried out on a VARIAN Cary Eclipse Fluorescence Spectrophotometer combined with Cary Eclipse Win FLR Software (Agilent, Vienna, Austria). The percentage of leakage was calculated as $I_{F}=\frac{\left(F-F_{0}\right)}{\left(F_{\max}-F_{0}\right)}$, where *F* is the measured fluorescence,$\;F_{0}$ is the initial fluorescence without peptide, and $F_{\max}$ is the fluorescence corresponding to 100% leakage achieved by the addition of 1% Triton X-100 [15,36].

#### 2.3.8. Phosphate Determination

To determine the phosphate content in the LUVs (nominal concentration: 20 mg/mL) for subsequent fluorescence measurement, the standard protocol by Broekhuyse [37] was taken as a basis. Lipid samples were heated first for about 1 min at 180 °C in an electrically heated metal block. To each tube, 0.4 mL acid mixture (conc. H_2_SO_4_/HClO_4_, 9:1 (*v*/*v*)) was added, and the mixture was heated for another 30 min at 180 °C. After cooling, 9.6 mL of an ANSA-molybdate mixture was added. ANSA-molybdate mixture was prepared by mixing of 11 mL reagent A (10.5 mM ANSA (1-amino-2-hydroxy-naphthalin-4-sulfonic acid), 0.7 mM Na_2_S_2_O_5_, 40 mM Na_2_SO_3_) with 250 mL reagent B (ammonium heptamolybdate tetrahydrate (0.26%)). After mixing, the tubes were placed in a sand bath at 90 °C for 20 min. The extinction of the cooled samples was measured on a Spectrophotometer Onda V-10 Plus at 830 nm. The PL concentration was calculated by using the calibration curve (3.2 mM KH_2_PO_4_ as phosphate-standard-solution containing 1—14 µg phosphor). In total, H_2_SO_4_ (96% (*v*/*v*)), Na_2_S_2_O_5_, and Na_2_SO_3_ were purchased from Carl Roth (Graz, Austria), HClO_4_ and ammonium heptamolybdate tetrahydrate (2.60 g/L) from Sigma-Aldrich, and ANSA and KH_2_PO_4_ (0.0997 mg/mL) from Merck (Vienna, Austria).

#### 2.3.9. Surface Charge Neutralization of Model Membranes and ζ-Potential Measurements

The ζ-potential measurements were carried out on a Zetasizer Nano (ZSP, Malvern Panalytical, Prager Electronics, Wolkersdorf, Austria) using disposable folded polycarbonate capillary cells (Malvern), according to the previously described procedure [38,39,40]. HEPES buffer was filtered by using a syringe filter with 0.02 µm pore size (AnotopTM, Sigma Aldrich, Vienna, Austria). In total, 50 µM of LUVs composed of POPG/TOCL or DMPG/TMCL at 80:20 molar ratio were mixed with the appropriate peptide amount that varied from 0.125 to 8 µM, which corresponds to 0.25 to 16 mol% peptides.

#### 2.3.10. Thermotropic Phase Behavior of Model Membranes and Differential Scanning Calorimetry (DSC)

DSC measurements were performed on binary mixture DMPG/TMCL (80:20 mol/mol) using a Nano-DSC high-sensitivity differential scanning calorimeter (Waters, Eschborn, Germany). Pure DMPG and TMCL were recorded as controls. Scans of 1 mg/mL lipid concentration were recorded at a constant heating rate of 0.5 °C/min. Data were analyzed using Launch NanoAnalyze. After normalization to the mass of PL and baseline correction, calorimetric enthalpies were calculated by integration of peak areas. The temperature of the peak maximum was taken as the main phase transition temperature.

## 3. Results

### 3.1. OP-145 and SAAP-148 Kill E. hirae in a Concentration and Time-Dependent Manner

First, we determined the antimicrobial activities of SAAP-148 and OP-145 against *E. hirae* during exposure for 5–120 min (Table 1). After 5 min, 0.4 µM SAAP-148 was necessary to kill 99.9% of bacteria, whereas an eightfold higher concentration of 3.2 µM was required in the case of OP-145 to induce the same effect. The time required for killing strongly depended on concentration. Lower concentrations of peptides, 0.2 µM and 0.1 µM of SAAP-148 and 1.6 µM of OP-145, induced cell death after 10 min. The most dramatic time-dependent effect was observed for OP-145 at 0.8 µM, where the activity was reduced to an incubation time of 60 min.

### 3.2. SAAP-148 Affects Membrane Integrity of E. hirae More Significantly Than OP-145

**Figure 2:**
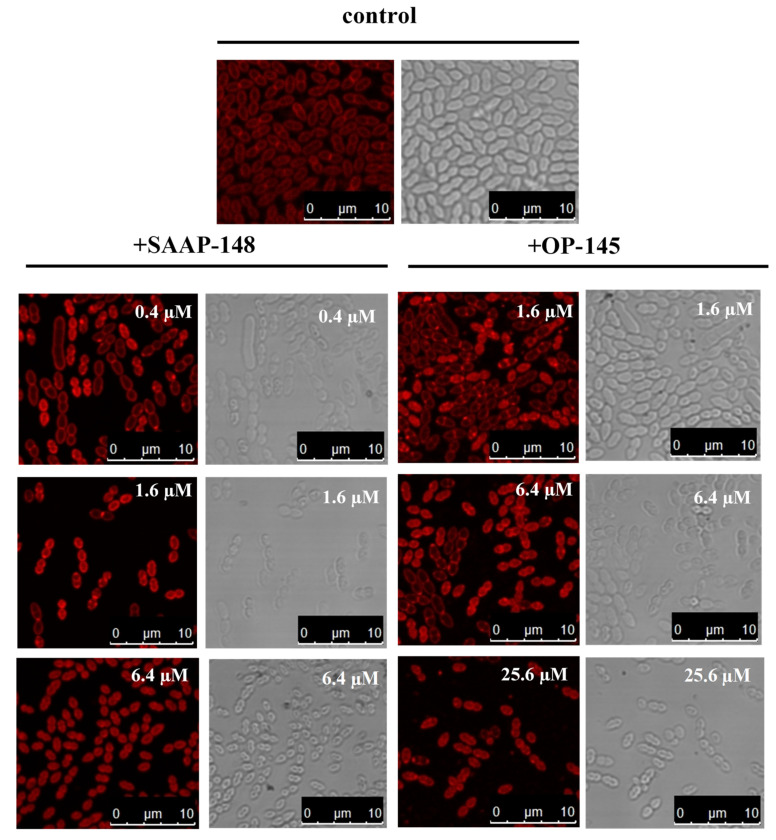
Fluorescence and bright-field images of *E. hirae* cells stained with Nile Red untreated and after incubation with indicated concentrations of OP-145 and SAAP-148.

Next, we stained *E. hirae* with the fluorescent membrane dye Nile Red in order to detect changes at the membrane of *E. hirae* exposed to our peptides (Figure 2). The staining of the bacterial membrane by Nile Red is sensitive to the polarity and fluidity of the membrane. The septum of dividing cells and areas around the connection zones of two cocci [41] are also intensely stained, as it is the case for diplococci *E. hirae* in our experiment.

Nile Red staining of untreated wild-type cells was more homogenous across the cell membrane compared with treatment with peptides. With increasing peptide concentration, Nile Red started to accumulate at different membrane areas and appeared first as visible foci in the membrane, before it markedly was detected in cytosol. This indicates that significant membrane damage occurred, thereby facilitating penetration of Nile Red into the cytosol. Those cells were markedly smaller than less affected cells indicating significant membrane leakage. In comparison with the diameter of wild-type cells of about 2 µm, SAAP-148-treated cells shrank to 0.98 µm at the highest applied concentration (6.4 µM), and OP-145-treated cells shrank to a diameter of 1.5 µm (25.6 µM). In conclusion, morphological effects on the membrane were more pronounced for SAAP-148 than for OP-145.

### 3.3. SAAP-148 Depolarizes E. hirae Cytoplasmic Membranes More Efficiently Than OP-145

**Figure 3:**
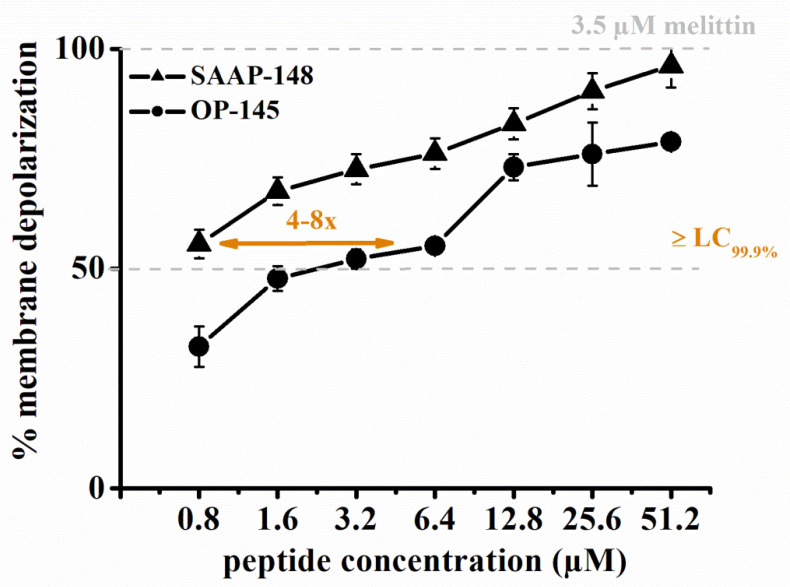
Effect of SAAP-148 and OP-145 on *E. hirae* membrane potential. Membrane depolarization of *E. hirae* at indicated peptide concentrations of SAAP-148 (triangles) and OP-145 (circles) in the range of 0.8 to 51.2 µM. Dashed line represents membrane depolarization induced by 3.5 µM melittin and set at 50% and 100%. Data points are averages of three independent experiments, with standard deviations indicated by error bars.

We used membrane-depolarization assays to detect peptide-induced changes in the membrane potential of *E. hirae*. To this end, we used the voltage-sensitive fluorescent dye DiSC_3_(5). DiSC_3_(5) is a cationic dye that translocates into the cytoplasmic membrane of energized cells and accumulates in the bilayer via self-association into a non-fluorescing oligomer [42]. If the membrane is depolarized, the dye will be released and its fluorescence de-quenched. Therefore, the depolarization of the membrane can be followed by the increase of the fluorescence intensity of DiSC_3_(5)-stained cell suspension. As shown in Figure 3, SAAP-148 was significantly more efficient in depolarizing *E. hirae* membranes than OP-145. To induce ~50% depolarization of *E. hirae* membranes required only 0.8 µM of SAAP-148, but 3.2–6.4 µM of OP-145. This is a 4-8-fold difference between the peptides.

### 3.4. SAAP-148 Permeabilizes E. hirae Cytoplasmic Membrane More Efficiently Than OP-145

Further, we tested OP-145 and SAAP-148 for their ability to permeabilize cytoplasmic membrane of *E. hirae*. For that purpose, we used flow-cytometric analysis and followed the influx of membrane-impermeable propidium iodide (PI) into the cytosol. In case of severe membrane damage, such as induced by AMPs, PI can penetrate into the cytosol, resulting in a fluorescence change upon binding to DNA and detection of PI-positive cells.

**Figure 4:**
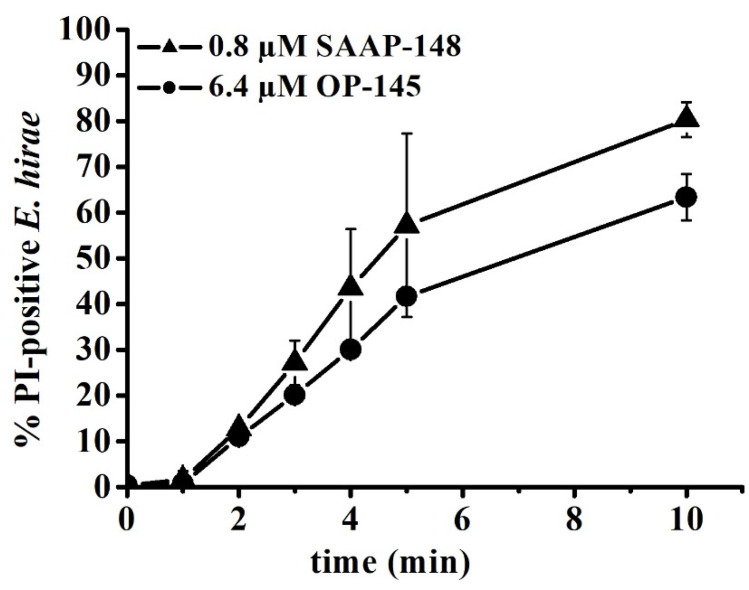
Membrane permeabilization of *E. hirae* by the peptides OP-145 and SAAP-148. The percentage of PI-positive *E. hirae* cells was calculated at different time intervals after the addition of 0.8 µM SAAP-148 and 6.4 µM OP-145. Results are averages of three independent experiments. Only the data for the two lethal concentrations are shown.

In order to compare the permeabilizing effects of the two peptides in the same time frame, PI entrance was followed upon exposure to peptides above their killing concentrations, that is, 0.8 µM SAAP-148 and 6.4 µM OP-145, over a period of 10 min (Figure 4). As expected, both peptides permeabilized the membrane of *E. hirae* in a time-dependent manner. Permeabilization started similarly for both peptides, but after ~3 min, it increased from 30% to 80% and from 20% to 60% of PI-positive cells for *E. hirae* exposed to SAAP-148 and OP-145, respectively. In accordance with the above results, this indicates that SAAP-148 induced a significantly stronger damage of the bacterial cytoplasmic membrane than OP-145.

### 3.5. SAAP-148 Interacts More Avidly with Membranes at the Molecular Level Than OP-145

In order to gain information on lipid-specific interactions of OP-145 and SAAP-148, model membrane systems composed of PG/CL resembling the membrane composition of *E. hirae* were used. The impact of the peptides on surface charge and thermotropic behavior and their membrane-disruption capability were assessed by ζ-potential measurements, DSC, and leakage assays.

**Figure 5:**
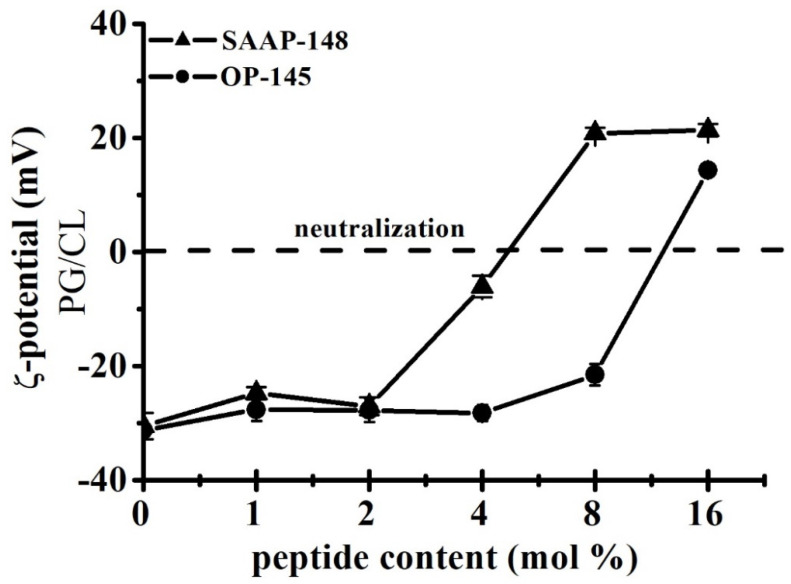
Neutralization of the surface charge of PG/CL membranes by SAAP-148 and OP-145. The **ζ-** potential measurements of PG/CL in the presence of SAAP-148 (triangles) and OP-145 (circles) at indicated concentrations ranging from 1 mol% to 16 mol%. Data are summary of three independent experiments. Measurements were performed on the POPG/TOCL mixture as well as on the DMPG/TMCL mixture. There was no difference between POPG/TOCL and DMPG/TMCL measurements in the presence of OP-145. Although a slight difference in concentration dependency is noticed for SAAP-148, the neutralization trend between POPG/TOCL and DMPG/TMCL is similar. Here, only measurements for POPG/TOCL are shown.

First, we performed ζ-potential measurements on PG/CL in the presence and absence of SAAP-148 and OP-145 (Figure 5). The ζ-potential of PG/CL assumed a value of –30 mV in the absence of peptide. The addition of the peptide changed the ζ-potential towards neutralization and led to an overcompensation at higher peptide concentrations, especially for SAAP-148. As expected, SAAP-148 is the peptide with the higher neutralization potential (~4 mol%) as compared with OP-145 (>8 mol%).

**Figure 6:**
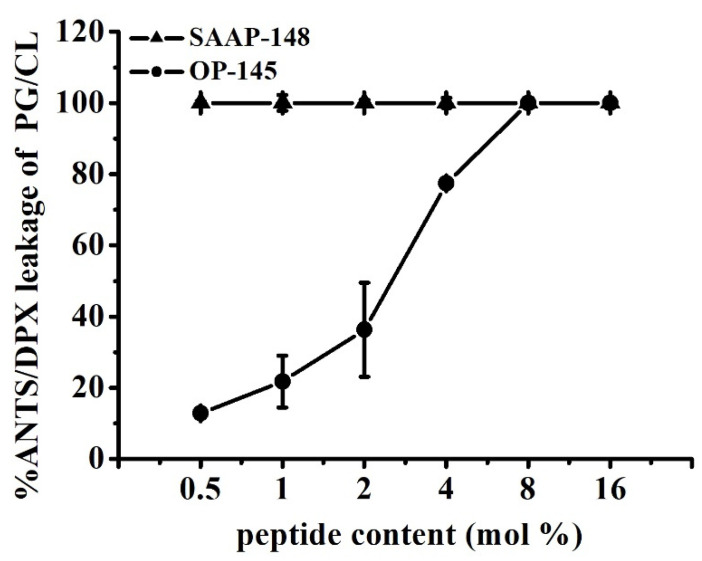
Permeability of PG/CL membranes induced by SAAP-148 and OP-145. Leakage of LUVs composed of PG/CL at indicated peptide concentrations of SAAP-148 (triangles) and OP-145 (circles) in the range of 0.5 mol% to 16 mol%. The full leakage is represented by 100% dye release upon addition of 1% (*v*/*v*) triton X-100. Data are the summary of three independent experiments.

Next, in analogy to ζ-potential experiments, we examined the permeability of the model membranes by performing fluorescence-based leakage experiments (Figure 6). The peptides were added in incremental amounts ranging from 0.5 mol% to 8 mol% to LUVs composed of PG and CL. The release of enclosed fluorescence dye molecules from PG/CL vesicles was observed already at the lowest concentration for both peptides tested (0.5 mol%). Full leakage was reached at 0.5 mol% SAAP-148, while 8 mol% OP-145 was needed for the same effect. This is a 16-fold difference between the peptides. By comparing these results with leakage data from pure PG membranes [3,15], it is evident that a lower peptide concentration of SAAP-148, but not of OP-145, is necessary for permeabilizing PG/CL membranes than for PG membranes. Although both peptides completely neutralized the surface charge of PG/CL membranes, disruption of these membranes occurred at concentrations lower than those required for neutralization.

**Figure 7:**
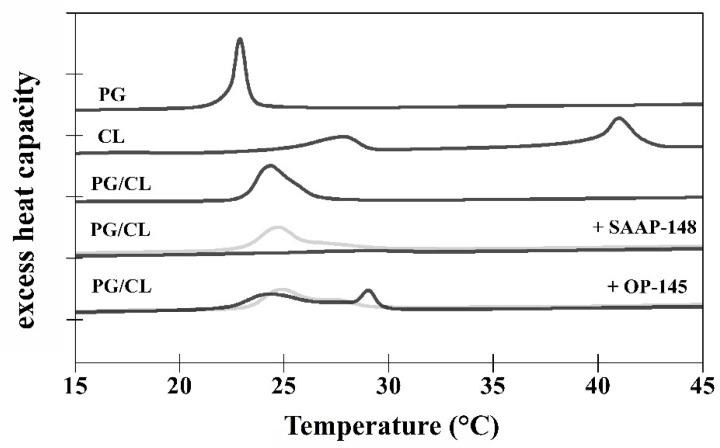
Thermotropic behavior of PG/CL membranes upon exposure to SAAP-148 and OP-145. DSC graphs show curves recorded for PG, CL, and PG/CL membranes and peptide-dependent curves for PG/CL in the presence of SAAP-148 and OP-145 as observed in heating scans. Grey lines indicate treatment with 1 mol%, black lines with 4 mol% peptide. For each sample, three heating and three cooling scans were performed between 1 °C and 75 °C with a scan rate of 0.5 °C/min. Here, the third heating scan of each measurement is shown, which is representative of all six scans.

To assess how the peptides OP-145 and SAAP-148 interact with the lipid membrane, DSC was performed using the model membranes PG/CL (Figure 7). Measurements were done before and after exposure to the peptides at concentrations of 1 mol% and 4 mol%. Transition temperatures and melting enthalpies are summarized in Table 2.

In accordance with the reported data [43,44], three well-separated phase transitions could be observed for CL. The low-temperature transition (*T*_low_) from the subsubgel phase (metastable gel phase with tilted fatty acids) to the lamellar gel phase occurred at 17.9 °C. The pre-transition temperature (*T*_pre_) from the lamellar gel to the ripple phase was at 27.9 °C. The main transition (*T*_m_) to the fluid phase occurred at 41.0 °C. PG [44] showed two transitions, the pre-transition at 12.0 °C and the main transition at 22.9 °C. The mixture of PG/CL [44] at a molar ratio of 80/20 revealed a phase transition temperature at 24.4 °C located near the temperature of pure PG. Very small pre- and low temperature transitions were detected for PG/CL. The two peptides had different disordering effects on the model membrane PG/CL. However, both peptides induced a slight shift of the main transition to higher temperature at 4 mol%. According to Lohner [45], a shift in transition temperature is a result of tighter lipid packing. This is observed, for example, for peptides inducing “quasi interdigitation” of the hydrocarbon chains within the bilayer, which has been shown for OP-145 [15] and also deduced for SAAP-148 [3] in the presence of PG membranes. Indeed, also for PG/CL, at a concentration of 1 mol%, SAAP-148 led to slightly increased transition temperature to 24.8 °C, whereas the total enthalpy of 10.1 kcal/mol was almost indistinguishable from that of untreated membranes (Δ*H*_m_ = 9.9kcal/mol). A broad shoulder underlying the main transition was also detected, which may result from the formation of small vesicular structures [45]. By increasing the peptide concentration of SAAP-148 to 4 mol%, the main transition disappeared and a transition could hardly be detected, which may correlate with the detected shoulder at lower peptide concentration. This transition at higher temperatures (28.3 °C) was characterized by an enthalpy of 2.1 kcal/mol. OP-145 showed a similar profile to SAAP-148, whereby the main transition was overlapping with a distinct broad transition at 1 mol% (*T*_m_ = 25.0 °C) being stronger pronounced at 4 mol%. Interestingly, this new phase transition resulted in an increase of the enthalpy and temperature shift to higher temperatures of 29.1 °C, in the direction of that from pure CL. This might be indicative for separation of the PG/CL membranes into peptide-enriched and poor CL domains. OP-145’s ability to induce the formation of lipid domains has previously been observed also for PG membranes [15].

## 4. Discussion

As part of the human microbiota, pathogenic Gram-positive *Enterococci* strains have attained important clinical significance as they can cause urinary-tract infections, endocarditis, and bacteremia [46]. In addition, enterococcal bacteria are on the list of the multidrug-resistant (MDR) strains with critical treatment that belong to the so-called ESKAPE (*Enterococcus faecium*, *Staphylococcus aureus*, *Klebsiella pneumoniae*, *Acinetobacter baumannii*, *Pseudomonas aeruginosa*, and *Enterobacter* species) panel [3]. *Enterococcus faecium* (10%) and *E. faecalis* (80%) species are the major enterococcal isolates in humans [47]. According to Dicpinigaitis et al. [48], infections with *E. hirae* have rarely been reported in humans, but they are not that uncommon in other mammals and in birds. Some cases have been described with *E. hirae* bacteremia associated with acute cholecystitis, acute pancreatitis, and septic shock, as well as endocarditis [47,48]. Both peptides, especially SAAP-148, show a very promising activity against these ESKAPE pathogens.

The question then arose which components build up the cell envelope of *Enterococci* and which target may be important for AMP’s interaction? Basically, the enterococcal membrane architecture follows the structure of other Gram-positive bacteria [2], with a single PL membrane surrounded by a thick cell wall composed of PGN and LTA/TA acid [49]. Both contribute largely to the overall negative charge of enterococcal cell envelopes. Due to presence of L-lysine with a missing carboxyl group, PGN also confers a single negative net charge. Gram-positive cytoplasmic membranes are negatively charged due to the presence of anionic PLs: mainly phosphatidylglycerol (PG), cardiolipin (CL), or lysyl-PG [2,21]. *E. hirae* also contains small amounts of glycolipids [27]. In addition, proteins, such as autolytic enzymes (e.g., murimidases), are associated with the cell wall [50] and are responsible for the degradation of PGN, while inhibiting cell-wall growth and cell division [51].

Taken together, on their way across the cell wall, AMPs might interact with several targets and end up also influencing autolytic degradation of PGN. In our previous work on OP-145, we clearly demonstrated that membrane permeabilization is not hindered by the presence of LTA and PGN [15], which is also true for SAAP-148 (data not shown). It seems plausible that peptides might induce cell wall autolysis as Nile Red staining of the *E. hirae* membranes in the presence of our AMPs first showed changes at the poles and the septum. It has already been reported that cell wall autolysis, cell division, and inhibition of cell growth of *B. subtilis* are related to bilayer defects and changes in membrane fluidity induced by the AMP cWFW [33]. Indeed, some changes in membrane fluidity and permeabilization have been observed in *B. subtilis* also treated with SAAP-148 [52]. However, with increasing concentrations of our peptides, the membrane could not resist changes in turgor, and *E. hirae* cells shrank to a considerably lower size. This also further points to peptide’s action at the cytoplasmic membrane.

Interestingly, staining of the membranes and cell shrinkage clearly showed concentration-dependent differences between peptides. Thus, SAAP-148 is not only more effective than OP-145 in killing *E. hirae,* but also in performing at the membrane level. This was further supported by the potent depolarization and permeabilization of the membrane through SAAP-148 action. For better comparison we summarized all effects as observed in experiments performed within 5 min exposure of the peptides to *E. hirae* (Table 3). After 5 min exposure to *E. hirae*, antimicrobial activity is 8-fold higher for SAAP-148 than for OP-145. Indeed, such a high difference was also observed for membrane activity. The clearest evidence for SAAP-148 superiority over OP-145 is reflected in the increase of membrane permeability, which is more than 16-fold in model membranes and >32 fold in *E. hirae*.

It is always a question if this potency can be ascribed to the higher positive net charge of SAAP-148 of +11, whereas OP-145 has a net charge of only +6. Thus, electrostatic interactions might be the dominant force in killing bacteria by SAAP-148 due to enhanced interaction with anionic lipid headgroups. Although SAAP-148 exhibited 16-fold higher membrane disruption potential than OP-145, our results from membranes composed of anionic PG/CL mixtures showed no evidence for electrostatic action during membrane disruption. Both peptides dose-dependently neutralized the surface of PG/CL membranes, but neutralization occurred at peptide concentrations higher than those required for leakage. At concentrations where membranes became completely leaky, the surface charge was only beginning to change. This suggests that the larger hydrophobic region of SAAP-148 might contribute to its superiority, which in particular might facilitate stronger partitioning and insertion into the membrane. Further, this may be indicative for a pore-forming mechanism or, at least, local accumulation of the peptide on a membrane, which might eventually result in the thinner membrane. In this case, no coverage of the membrane surface by the peptides is necessary for induction of the transmembrane pore formation and can emerge below the threshold concentration [20]. The same would be true if peptides align parallel to the membrane surface, inducing a void in the hydrophobic core, which can be compensated by chain interdigitation. Indeed, results obtained from DSC measurements (Figure 7, Table 2) hint for peptide induced thinning of the membrane. Usually, membrane thinning is observed by peptides exhibiting an ideal lateral amphipathicity [20], which is the case for our peptides. Such effects were observed on PG/CL, as well as PG-only membrane for both peptides, which do not differ in their amphipathic character (Figure 1). This kind of action contributes significantly to the destabilization of the membranes, however, being again more pronounced for SAAP-148. The induction of “quasi-interdigitated” membrane domains is accompanied by a slight increase in phase transition temperature and usually ~20% higher enthalpy of the membrane. Such a behavior was observed at 4 mol% OP-145 or 2 mol% SAAP-148 on PG [3,15]), and 1 mol% OP-145 or SAAP-148 on PG/CL membranes. The fact that stronger effects were observed on PG/CL than on PG-only membrane (see our previous work for both, OP-145 [15] and SAAP-148 [3]), points also to some differences in structure of the peptides and membranes, which might be related to other differences than the larger hydrophobic angle and smaller ∆G of SAAP-148. Both membranes are anionic, whereby CL, in addition, may induce membrane curvature and may undergo hydrogen bonding with the peptides side chain groups. Membrane curvature might affect peptides’ insertion into the membrane, and finally result in different local deformation of the bilayer [20]. Given the fact that electrostatic interactions are not necessary for induction of membrane thinning, most probably hydrogen bonding activity to CL may potentiate the actions of the peptides. Here again, more favorable contacts to CL-head groups for SAAP-148 due to the presence of Q18 and Y15 in the hydrophobic region. As such contact sides are missing in OP-145 sequence, OP-145 might not interact efficiently with CL, which also can explain the remarkable phase separation of PG/CL (Figure 7) and similar membrane disruption potential compared to that induced on PG membrane [15]. Besides the larger hydrophobic face, SAAP-148 bears W5 and W16 in close proximity to the hydrophobic/hydrophilic interface of the peptide. It is known that W resides preferentially in the membrane interface [53] and has a significant role in the interfacial membrane anchoring. This further points to differences in insertion of both peptides into the membrane.

Hence, SAAP-148 might also show higher activity towards eukaryotic membranes. Although the therapeutic potential of SAAP-148 is very promising, it induces some hemolysis of red blood cells at higher concentrations [54]. However, the tolerated 5% hemolysis does not exceed at the concentration where SAAP-148 exhibits antimicrobial activity. So far, SAAP-148 was well tolerated in topical treatments on the murine skin in vivo and human skin, but was cytotoxic at relatively low concentrations in human skin cells in 2D culture [9]. A similar scenario also holds for OP-145. Its lack of in vivo toxicity was demonstrated in guinea pigs, rabbits, and rats [13] as well as in clinical trials on patient with chronic otitis media [16], but it showed some cytotoxicity against human cells [15]. Nevertheless, these observations emphasize the complexity at the cellular level, and hence it is one of the biggest challenges in the antimicrobial peptide research to develop peptides with a high therapeutic index.

In summary, our results demonstrate that OP-145 and SAAP-148 are effective in killing *E. hirae*, with SAAP-148 being effective at a significantly lower concentration than OP-145. Both peptides target the cytosolic membrane of *E. hirae*, with SAAP-148 again inducing significant effects on the membrane at markedly lower concentrations. The same holds true for the capability of the two peptides to disrupt membranes. Thus, we can conclude that the potency of SAAP-148 to kill bacteria derives from its superior ability to disrupt the bacterial membrane.

## Figures and Tables

**Table 1:** Antimicrobial activity of SAAP-148 and OP-145 against *E. hirae*. In total, 1 × 10^6^ CFU/mL of *E. hirae* was exposed to 0.1–0.4 µM of SAAP-148 and 0.8–3.2 µM of OP-145. After 5, 10, 20, 30, 60, and 120 min of incubation, numbers of viable bacteria were determined by counting CFUs. Lethal concentrations (LC_99.9%_) at which peptides killed 99.9% cells are indicated in grey. Data are averages of three independent experiments.

Peptide	Concentration (µM)	Time (min)					
		5	10	20	30	60	120
**SAAP-148**	0.1						
	0.2						
	0.4						
**OP-145**	0.8						
	1.6						
	3.2						

**Table 2:** Thermodynamic parameters (low-temperature transition, pretransition, and main transition temperature (T_low_/T_pre_/T_m_) and corresponding enthalpies (∆H_low_/∆H_pre_/∆H_m_)) of PG, CL, and PG/CL membranes in the presence and absence of the 1 mol% and 4 mol% SAAP-148 and OP-145.

	*T*_low_	∆*H*_low_	*T*_pre_	∆*H*_pre_	*T*_m_	∆*H*_m_
	(°C)	(kcal/mol)	(°C)	(kcal/mol)	(°C)	(kcal/mol)
**PG**			12.0	1.0	22.9	7.9
**CL**	17.9	4.7	27.9	3.8	41.0	17.3
**PG/CL**					24.4	9.9
**PG/CL + SAAP-148**						
**1 mol%**					24.8 ^#^	10.1 *
**4 mol%**					28.3	2.1
**PG/CL + OP-145**						
**1 mol%**					25.0 ^#^	10.1 *
**4 mol%**					24.3; 29.1	16.1 *

* Total enthalpy. Because of peak overlap, only the total enthalpy could be determined accurately. ^#^ Transition with overlapping shoulder.

**Table 3:** Comparison of peptide activities after 5 min of incubation.

	*E. hirae*	*E. hirae* Membrane		Model Membranes	
	99.9% killing	half-maximal depolarization	half-maximal permeability	maximal surface neutralization	maximal permeability
**SAAP-148**	0.4 µM	0.8 µM	0.8 µM	>4 mol%	0.5 mol%
**OP-145**	3.2 µM	6.4 µM	>25.6 µM	>8 mol%	8 mol%

## Data Availability

The data presented in this study are available on request from the corresponding author.

## References

[1] Hancock R.E.W., Sahl H.-G. (2006). Antimicrobial and host-defense peptides as new anti-infective therapeutic strategies. Nat. Biotechnol..

[2] Malanovic N., Lohner K. (2016). Antimicrobial Peptides Targeting Gram-Positive Bacteria. Pharmaceuticals.

[3] de Breij A., Riool M., Cordfunke R.A., Malanovic N., de Boer L., Koning R.I., Ravensbergen E., Franken M., van der Heijde T., Boekema B.K. (2018). The antimicrobial peptide SAAP-148 combats drug-resistant bacteria and biofilms. Sci. Transl. Med..

[4] Malanovic N., Marx L., Blondelle S.E., Pabst G., Semeraro E.F. (2020). Experimental concepts for linking the biological activities of antimicrobial peptides to their molecular modes of action. Biochim. Biophys. Acta Biomembr..

[5] Lohner K. (2009). New strategies for novel antibiotics: Peptides targeting bacterial cell membranes. Gen. Physiol. Biophys..

[6] Boman H.G. (2003). Antibacterial peptides: Basic facts and emerging concepts. J. Intern. Med..

[7] Wang G., Li X., Wang Z. (2016). APD3: The antimicrobial peptide database as a tool for research and education. Nucleic Acids Res..

[8] Kumar P., Kizhakkedathu J.N., Straus S.K. (2018). Antimicrobial Peptides: Diversity, Mechanism of Action and Strategies to Improve the Activity and Biocompatibility In Vivo. Biomolecules.

[9] Dijksteel G.S., Ulrich M.M.W., Middelkoop E., Boekema B.K.H.L. (2021). Review: Lessons Learned From Clinical Trials Using Antimicrobial Peptides (AMPs). Front. Microbiol..

[10] Ridyard K.E., Overhage J. (2021). The Potential of Human Peptide LL-37 as an Antimicrobial and Anti-Biofilm Agent. Antibiotics.

[11] Haisma E.M., de Breij A., Chan H., van Dissel J.T., Drijfhout J.W., Hiemstra P.S., El Ghalbzouri A., Nibbering P.H. (2014). LL-37-Derived Peptides Eradicate Multidrug-Resistant Staphylococcus aureus from Thermally Wounded Human Skin Equivalents. Antimicrob. Agents Chemother..

[12] Nibbering P.H., Göblyös A., Adriaans A.E., Cordfunke R.A., Ravensbergen B., Rietveld M.H., Zwart S., Commandeur S., van Leeuwen R., Haisma E.M. (2019). Eradication of meticillin-resistant Staphylococcus aureus from human skin by the novel LL-37-derived peptide P10 in four pharmaceutical ointments. Int. J. Antimicrob. Agents.

[13] Nell M.J., Tjabringa G.S., Wafelman A.R., Verrijk R., Hiemstra P.S., Drijfhout J.W., Grote J.J. (2006). Development of novel LL-37 derived antimicrobial peptides with LPS and LTA neutralizing and antimicrobial activities for therapeutic application. Peptides.

[14] Ming L., Huang J.-A. (2017). The Antibacterial Effects of Antimicrobial Peptides OP-145 against Clinically Isolated Multi-Resistant Strains. Jpn. J. Infect. Dis..

[15] Malanovic N., Leber R., Schmuck M., Kriechbaum M., Cordfunke R.A., Drijfhout J.W., de Breij A., Nibbering P.H., Kolb D., Lohner K. (2015). Phospholipid-driven differences determine the action of the synthetic antimicrobial peptide OP-145 on Gram-positive bacterial and mammalian membrane model systems. Biochim. Biophyica Acta.

[16] Peek N.F.A.W., Nell M.J., Brand R., Jansen-Werkhoven T., van Hoogdalem E.J., Verrijk R., Vonk M.J., Wafelman A.R., Valentijn A.R.P.M., Frijns J.H.M. (2020). Ototopical drops containing a novel antibacterial synthetic peptide: Safety and efficacy in adults with chronic suppurative otitis media. PLoS ONE.

[17] Scheper H., Wubbolts J.M., Verhagen J.A.M., de Visser A.W., van der Wal R.J.P., Visser L.G., de Boer M.G.J., Nibbering P.H. (2021). SAAP-148 Eradicates MRSA Persisters Within Mature Biofilm Models Simulating Prosthetic Joint Infection. Front. Microbiol..

[18] Oren Z., Lerman J.C., Gudmundsson G.H., Agerberth B., Shai Y. (1999). Structure and organization of the human antimicrobial peptide LL-37 in phospholipid membranes: Relevance to the molecular basis for its non-cell-selective activity. Biochem. J..

[19] Wildman K.A.H., Lee D.-K., Ramamoorthy A. (2003). Mechanism of Lipid Bilayer Disruption by the Human Antimicrobial Peptide, LL-37. Biochemistry.

[20] Lohner K. (2017). Membrane-active Antimicrobial Peptides as Template Structures for Novel Antibiotic Agents. Curr. Top. Med. Chem..

[21] Malanovic N., Lohner K. (2016). Gram-positive bacterial cell envelopes: The impact on the activity of antimicrobial peptides. Biochim. Biophys. Acta Biomembr..

[22] Sevcsik E., Pabst G., Jilek A., Lohner K. (2007). How lipids influence the mode of action of membrane-active peptides. Biochim. Biophys. Acta..

[23] Sevcsik E., Pabst G., Richter W., Danner S., Amenitsch H., Lohner K. (2008). Interaction of LL-37 with Model Membrane Systems of Different Complexity: Influence of the Lipid Matrix. Biophys. J..

[24] Sancho-Vaello E., Gil-Carton D., François P., Bonetti E.-J., Kreir M., Pothula K.R., Kleinekathöfer U., Zeth K. (2020). The structure of the antimicrobial human cathelicidin LL-37 shows oligomerization and channel formation in the presence of membrane mimics. Sci. Rep..

[25] Scheenstra M.R., van den Belt M., Tjeerdsma-van Bokhoven J.L.M., Schneider V.A.F., Ordonez S.R., van Dijk A., Veldhuizen E.J.A., Haagsman H.P. (2019). Cathelicidins PMAP-36, LL-37 and CATH-2 are similar peptides with different modes of action. Sci. Rep..

[26] Dorschner R.A., Lopez-Garcia B., Peschel A., Kraus D., Morikawa K., Nizet V., Gallo R.L. (2006). The mammalian ionic environment dictates microbial susceptibility to antimicrobial defense peptides. FASEB J..

[27] Boaretti M., Canepari P. (1995). Identification of daptomycin-binding proteins in the membrane of Enterococcus hirae. Antimicrob Agents Chemother.

[28] Shen Y., Maupetit J., Derreumaux P., Tufféry P. (2014). Improved PEP-FOLD Approach for Peptide and Miniprotein Structure Prediction. J. Chem. Theory Comput..

[29] Thevenet P., Shen Y., Maupetit J., Guyon F., Derreumaux P., Tuffery P. (2012). PEP-FOLD: An updated de novo structure prediction server for both linear and disulfide bonded cyclic peptides. Nucleic Acids Res..

[30] Beaufays J., Lins L., Thomas A., Brasseur R. (2012). In silico predictions of 3D structures of linear and cyclic peptides with natural and non-proteinogenic residues. J. Pept. Sci..

[31] Snider C., Jayasinghe S., Hristova K., White S.H. (2009). MPEx: A tool for exploring membrane proteins. Protein Sci..

[32] White S.H., Wimley W.C. (1999). MEMBRANE PROTEIN FOLDING AND STABILITY: Physical Principles. Annu. Rev. Biophys. Biomol. Struct..

[33] Scheinpflug K., Krylova O., Nikolenko H., Thurm C., Dathe M. (2015). Evidence for a Novel Mechanism of Antimicrobial Action of a Cyclic R-,W-Rich Hexapeptide. PLoS ONE.

[34] Wolinski H., Kohlwein S.D. (2015). Microscopic and spectroscopic techniques to investigate lipid droplet formation and turnover in yeast. Methods Mol. Biol..

[35] Zweytick D., Japelj B., Mileykovskaya E., Zorko M., Dowhan W., Blondelle S.E., Riedl S., Jerala R., Lohner K. (2014). N-acylated peptides derived from human lactoferricin perturb organization of cardiolipin and phosphatidylethanolamine in cell membranes and induce defects in Escherichia coli cell division. PLoS ONE.

[36] Zweytick D., Deutsch G., Andrä J., Blondelle S.E., Vollmer E., Jerala R., Lohner K. (2011). Studies on lactoferricin-derived Escherichia coli membrane-active peptides reveal differences in the mechanism of N-acylated versus nonacylated peptides. J. Biol. Chem..

[37] Broekhuyse R.M. (1968). Phospholipids in tissues of the eye. I. Isolation, characterization and quantitative analysis by two-dimensional thin-layer chromatography of diacyl and vinyl-ether phospholipids. Biochim. Biophys Acta.

[38] Freire J.M., Domingues M.M., Matos J., Melo M.N., Veiga A.S., Santos N.C., Castanho M.A.R.B. (2011). Using zeta-potential measurements to quantify peptide partition to lipid membranes. Eur. Biophys. J..

[39] Domingues M.M., Silva P.M., Franquelim H.G., Carvalho F.A., Castanho M.A.R.B., Santos N.C. (2014). Antimicrobial protein rBPI21-induced surface changes on Gram-negative and Gram-positive bacteria. Nanomedicine.

[40] Malanovic N., Ön A., Pabst G., Zellner A., Lohner K. (2020). Octenidine: Novel insights into the detailed killing mechanism of Gram-negative bacteria at a cellular and molecular level. Int. J. Antimicrob. Agents.

[41] Koch A.L. (2013). Bacterial Growth and Form.

[42] te Winkel J.D., Gray D.A., Seistrup K.H., Hamoen L.W., Strahl H. (2016). Analysis of Antimicrobial-Triggered Membrane Depolarization Using Voltage Sensitive Dyes. Front. Cell Dev. Biol..

[43] Prenner E.J., Lewis R.N.A.H., Kondejewski L.H., Hodges R.S., McElhaney R.N. (1999). Differential scanning calorimetric study of the effect of the antimicrobial peptide gramicidin S on the thermotropic phase behavior of phosphatidylcholine, phosphatidylethanolamine and phosphatidylglycerol lipid bilayer membranes. Biochim. Biophys. Acta (BBA) Biomembr..

[44] Benesch M.G., Lewis R.N., McElhaney R.N. (2015). On the miscibility of cardiolipin with 1,2-diacyl phosphoglycerides: Binary mixtures of dimyristoylphosphatidylglycerol and tetramyristoylcardiolipin. Biochim. Biophys. Acta.

[45] Lohner K., Bastos M. (2015). DSC Studies on the Modulation of Membrane Lipid Polymorphism and Domain Organization by Antimicrobial Peptides. Biocalorimetry: Foundations and Contemporary Approaches.

[46] Fiore E., van Tyne D., Gilmore M.S. (2019). Pathogenicity of Enterococci. Microbiol. Spectr..

[47] Anghinah R., Watanabe R.G.S., Simabukuro M.M., Guariglia C., Pinto L.F., Gonçalves D.C.d.M.E. (2013). Native Valve Endocarditis due to Enterococcus hirae Presenting as a Neurological Deficit. Case Rep. Neurol. Med..

[48] Dicpinigaitis P.V., de Aguirre M., Divito J. (2015). Enterococcus hirae Bacteremia Associated with Acute Pancreatitis and Septic Shock. Case Rep. Infect. Dis..

[49] Hancock L.E., Murray B.E., Sillanpää J., Gilmore M.S., Clewell D.B., Ike Y., Shankar N. (2014). Enterococcal Cell Wall Components and Structures. Enterococci: From Commensals to Leading Causes of Drug Resistant Infection.

[50] Shockman G.D., Martin J.T. (1968). Autolytic enzyme system of Streptococcus faecalis. IV. Electron microscopic observations of autolysin and lysozyme action. J. Bacteriol..

[51] Shockman G.D. (1992). The autolytic (‘suicidase’) system of Enterococcus hirae: From lysine depletion autolysis to biochemical and molecular studies of the two muramidases of Enterococcus hirae ATCC 9790. Fems. Microbiol. Lett..

[52] Liu S., Brul S., Zaat S.A.J. (2021). Isolation of Persister Cells of Bacillus subtilis and Determination of Their Susceptibility to Antimicrobial Peptides. Int. J. Mol. Sci..

[53] Yau W.-M., Wimley W.C., Gawrisch A.K., White S.H. (1998). The Preference of Tryptophan for Membrane Interfaces. Biochemistry.

[54] Li S., She P., Zhou L., Zeng X., Xu L., Liu Y., Chen L., Wu Y. (2020). High-Throughput Identification of Antibacterials Against *Pseudomonas aeruginosa*. Front. Microbiol..

